# The Student and Young Doctor1Introductory lecture to the Sixtieth Annual Course of Medicine at the University of Buffalo, September 25, 1905.

**Published:** 1905-11

**Authors:** George F. Cott

**Affiliations:** Buffalo, N. Y.; Clinical Professor of Otology, University of Buffalo, 85 North Pearl Street


### BUFFALO MEDICAL JOURNAL.

VOL. LXI. NOVEMBER, 1905. NO. 4

### ORIGINAL COMMUNICATIONS.

### The Student and Young Doctor.[1]

BY GEORGE F. COTT, M. D., Buffalo, N. Y.
Clinical Professor of Otology, University of Buffalo.

WHEN the dean invited me to address you upon this the beginning of the sixtieth year of this university, I confess I was at a loss concerning the subject. I am not expected to attain to the sublimity of last year's silver-tongued orator, I am sure, or I would not have been asked at all. The subject also has been left to my own discretion. Two years ago the address related to the conduct of the young physician; tonight I would like to include the freshman. You are aware, no doubt, that the course in this college has been extended to eight months; however, no more subjects have been added, and the hours of study made shorter, thus giving the student ample time for preparation. The percentage in examination no doubt will be raised above 70, thereby keeping the student thoroughly interested.

The way of the medical student is not along a path of roses; on the contrary, it is strewn with thorns and thistles. If many of the aspirants for medical honors knew the intricacies of the labyrinth from which they expect to emerge four years hence, they would engage in other pursuits. The Lord is kind, however, to the ambitious and guides them safely over the rocky trail.

Medicine is as full of discoveries as astronomy, electricity and the other sciences, and the world at large knows as little of its remarkable developments in this line as does the Christian Scientist of zymotic diseases.

You are tonight taking the first step in a dignified profession which has more responsibilities for the conscientious physician than your imagination can conceive. It behooves you, therefore, to master your work thoroughly. Never worry about the future; do your work well today and tomorrow will take care

1. Introductory lecture to the Sixtieth Annual Course of Medicine at the University of Buffalo, September 25, 1905.

of itself. It is not at all necessary to over-crowd your brain, in fact medical students seldom die of overwork. Nevertheless, in order to become proficient men of medicine, hours of work and hours of play must interchange. Out-door exercise is quite essential to proper mental development, therefore Buffalo University ought to have a good baseball club and the best football team in Western New York, and above all, during the winter months, a gymnasium ought frequently to be patronized, and this should be made obligatory. But alas! where is the gymnasium? The faculty cannot provide one from its funds and there is no endowment for that purpose.

There is at present a movement on foot to erect from this medical nucleus a great university that our children may become proficient in the arts and sciences here, instead of getting a degree elsewhere. A most commendable project.

But who are the men who have undertaken this stupendous task? Our rich citizens? By no means as yet, but men with big hearts and comparatively meagre purses. The common notion goes abroad that the seventy-five teachers of this institution absorb all the money which is paid for tuition; why not take that money for university extension? The teachers who receive pay are about five per cent. or less and these devote all their time to the work and have therefore very little, if any, outside practice. All the rest give their time gratis. The money received from students is all absorbed in maintaining the college. And I will state right here that this college is most thoroughly equipped with all scientific apparatus, making it compare favorably with any institution of its kind in the country. Furthermore, the University of Buffalo has graduated men who stand high in the councils of the profession. Its graduates rank second in successful examinations of the State board, and all this without any endowment. Does not this school deserve credit for all the good it has accomplished during its 60 years of existence? Does it not merit the ardent support of its alumni? Is it not an honor to this city and does it not therefore, deserve the support of the citizens of wealth in this community?

The method adopted by our vice-chancellor will bear abundant fruit and in a very few years there will be a complete University of Buffalo, a towering monument to the energetic and indefatigable exertions of its founders, and when complete it will bring thousands upon thousands of dollars to this city and keep other thousands at home which are now spent abroad. Of course it will take a great deal of money to maintain a university and this must be acquired in some way, but time will also solve that problem.

At the recent meeting of the American Medical Association at Portland, Oregon, the committee in charge of medical education, all men learned and of wide experience, reported as follows: One of the chief functions of the American Medical Association should be the elevation of medical education so as to make it as high as in any country in the world. Our position as a civilising power and in commerce and in arts and sciences demands this of American medicine. Medical studies should consist of five years after having passed preliminary examinations, the first year to consist of physics, chemistry and biology either taken in a school of liberal arts or medical college. Then four years of pure medical work and finally the sixth year as interne at a hospital or dispensary. Most men would then be 27 or 28 years of age. This, however, gives the candidate the doctorate degree only but does not entitle him to practice until he has passed an examination satisfactory to the State board. In an arts school where medicine is a department, a full course of four years is unfair to the medical student; he would be little less than 30 years old when he emerged. As one young man remarked recently, "I must go to school one-half of my life to learn how to take care of the other half."

Huxley said: "That man, I think, has a liberal education whose body has been so trained in youth that it is the ready servant of the will; of a tender conscience and one who has learned to love all beauty, whether of nature or of art, to hate all vileness, and to esteem others as himself."

After all it is not so much the amount of knowledge one acquires as the proper application of such knowledge. To become a successful practitioner requires far more than book learning. One must also have tact, good habits, a good share of business knowledge and above all common sense. Occasionally a young doctor who has never been used to handling his own money and who receives perhaps $100 per month the first year of his practice, will soon find his expense account greater than his income and gradually his bills go unpaid and often he falls by the way, finally to make his exit by morphine, cocaine, or the whiskey route. Give me the man whose first dollar looks the size of a cartwheel; he usually succeeds. It is quite apropos here to remind you that the sheepskin does not make the man; you do not know it all. Look around and you will notice all about you men who are your betters. You work away some years then have another look; you will still find yourself near the lower round of the ladder. But keep right on plugging; there is plenty of room at the top. It may look like an everlasting job, but bear in mind the good men whom you have looked up to with envy will gradually be gathered unto their fathers, then others will envy you. The vast majority of medical men remain good, poor or indifferent practitioners; for such the future seems to be a closed book. They take no interest in medical meetings, shun society and believe themselves ostracised by the profession at large. Such men believe opportunity never comes their way, forgetting at the same time that man often creates his own opportunity. Bear in mind there are 80,000,000 of people in this country who never did you a mean thing.

Sir Isaac Newton was considered rather dull at school and therefore was made the butt for the bullies, until one day he walloped the biggest one. That gave him a glimpse of his power and he straightway set to work to vanquish mentally those whom he combatted successfully physically. This he accomplished within a few months. Later, in speaking of those who had benefited him most he placed first on the list the boy he had whipped and said: "Our enemies are quite as neccessary as our friends."

The good man never gauges his success by the number of dollars he accumulates. Show me a wealthy doctor and I will show you one who spends very little time in his library. Chasing the dollar belongs to the commercial doctor only. True, we have wealthy men in the profession who are also great lights among men, but they came in possession of money by way of marriage or inheritance, rarely from the practice of medicine.

This school is small in comparison with many others but it is not always the large university which has for its teachers the greatest scholars. To meet the world-famed scientist, Ernest Heckel, one must visit Jena, a small town in Germany of perhaps 10,000 inhabitants, his students seldom number 50, yet every word he reads to his boys is eagerly appropriated throughout the world. Amherst has David P. Todd, the greatest astronomer of the New World. Ruskin read his best things in a little hall at Coniston to a dozen people. Morris's audiences at Hammersmith were less than 100. Kant, the great thinker, up to his 70th year was never more than six miles from his birthplace, yet he gave the world everlasting treasures. These men could have their choice among the greatest schools but nothing could lure them from their little towns.

Large universities have very little to do with the making of great men, but rather persistent application.

Bulwer Lytton said: "There is but one philosophy (though there are a thousand schools), and its name is Fortitude."

Darwin in his autobiography says, "Personally I never had much ambition, but when at college I felt that I must work."

Darwin's father wanted him to become a clergyman, "for", said he, "the boy is good for nothing else." Dr. Darwin lived long enough to see his son England's foremost scientist.

Rudolph Virchow, who promulgated his theory of cellular pathology over 50 years ago, worked incessantly until his death at the age of 81. His light was seen burning night after night until after three o'clock, yet he was at his lectures again at six in the morning. He was supposed to sleep seldom more than two out of the twenty-four hours.

I heard him at the Berlin polyclinic in '95, a little man about five feet four inches in height, thin and shriveled, but a power of physical endurance.

Joseph O'Dwyer, the inventor of intubation, labored industriously for years, operating upon hundreds of children in order to discover a method whereby he could save the lives of those afflicted with the dread destroyer, diphtheria. He died at the early age of fifty-five.

Pasteur, who discovered the germ which destroyed the silk worm in France, and thereby laid the foundation of bacteriology, worked unceasingly until his death at the age of seventy.

Manuel Garcia, who fifty years ago discovered the laryngoscope, whereby the interior of the larynx could be observed during phonation, is active as ever to-day. He celebrated his one hundredth birthday April 17, 1905, upon which occasion he received decorations from three kings.

I might mention a long list of the world's greatest men, their poverty, privation and other hardships they had to undergo, but let this suffice.

No man becomes great without hard, hard work; nor is age any barrier, in fact most of our eminent men are past middle age. The medical man who wishes to tower above his colleagues must work while they sleep. His greatest ambition is to do more and more. Money is not a requisite, but ambition. President Hadley of Yale says: the boy who gets the most out of college is the boy who works his way through.

Garfield, a poor boy, was a mule driver on the canals at the age of sixteen. He died president of the United States. Lincoln was too poor to own a lamp so he lay down before a wood fire to study. Cornelius Vanderbilt the first, used to run a ferry-boat as a boy and died a millionaire.

Truly the one philosophy is fortitude.

Great wealth is not usually due to the brains of those who possess the money. This hardly applies to the medical man for he seldom becomes a frenzied financier.

The laboratory, the great workshop of industrious students, gives up its secrets to make possible Standard Oil monopoly, amalgamated copper and the great sugar trust. Were it not for the laboratory Rockefellerism would be impossible.

The State Laboratory located in this city is but a drop in the bucket of science, yet it bids fair to discover sooner or later the cause of that terrible destroyer, cancer. But people at large have not the slightest idea of the work entailed to convince our state legislative body that it takes a few thousands of dollars annually to carry on the work. Many years are consumed before a final result is obtained. The scientist and the politician make poor bed fellows.

Such institutions should be made absolute, maintained by the State, and perpetuated by constitutional law. In Europe all such institutions are supported by the government, and for that reason the continent has so great a reputation with our American students.

Great praise is due the American physician for his unselfish work in developing the science and elaborating the arts of medicine without government support. Professor Ewald, while visiting this country several years ago, said: "Students from all over the world now flock to Europe, but I believe and hope that in the not distant future they will come to this country instead, for the American physician is making remarkable progress."

Now, young men, permit me to observe, that you will in your professional life have to contend with other creatures besides mere men of scientific medicine. You will find sharpers and fakirs on all sides, from pleasant purgative pellets and pink pills for pale people, to men cured to stay cured, from Lydia Pinkham's hair restorer for bald-headed women, to a golden medical observatory. All these medical mendicants, composed of shrewd but ignorant individuals, are trying by legislative enactment or otherwise to become legalised charlatans.

One of the recent fake introductions is osteopathy. Massage has been practised many years but the income was very meagre until some fertile brain conceived the idea to give it a new name which, though without meaning, would nevertheless appeal to the gullable people of which this country is overflowing, and adding thereto that in every case the ailment is due to the dislocation of a vertebra. All concerning this new cult may be acquired for a stipulated sum, either by letter or a few months attendance at one of their numerous mushroom colleges. As far as I know there is no antipathy against these men and women practising massage any more than against a man making use of homeopathy. We deny them the right however, to disgrace the profession by assuming the title of doctor. But that is really all they want, without however, acquiring it according to law, established custom and usage. There is no objection to anyone calling himself Doctor of Osteopathy after he has fully met the requirements of the present law in this state, which is amply sufficient to protect all who have passed the existing board of examiners. We hope the profession will always have sufficient able cases those involved would speedily die of inanition, because there are so few such.

The question might occur to some of you, what makes these fakirs possible? and I would answer: the average physician gives drugs; which is very simple and requires no particular physical exertion. But he often forgets other measures more appropriate, hence the chief fakir of a cult steps in and supplies the omission. Is there no good in osteopathy, Kneipp bare-foot wet grass cure, Christian Science, cancer-cure and all the other kinds of quackery? Certainly there is, but if applied only to suitable cases those involved would speedily die of inanition, there are so few.

Therefore, let me remind you that all unscientific practices are not for doing good or for relieving suffering humanity but solely for the making of money, for lining the pocketbooks of lazy vagabonds who know that nine-tenths of the American people are willing to pay for being humbugged and pay well too.

Students before graduating often aspire to specialism. Do not think of it for some years to come; then if so inclined, select the one which to you presents the *most* difficulties and make it your life's work. All the specialists in your particular choice will not welcome you with open arms; some, on the contrary may put barriers in your way and possibly belittle your work to patients. Let this not hinder, but spur you on to better work until, by demonstration, you have won them over. If you have sufficient genius to introduce new methods to relieve suffering humanity or invent instruments superior to those in use, be a true physician and give freely to those who have not talent equal to yours. It is not wealth you are after but the relief of the suffering. Paré, the greatest of French surgeons in the 16th century, who exerted a mighty influence in surgical Europe, said: "For my part I have dispensed liberally to everybody the gifts that God has conferred upon me and I am none the worse for it; just as the light of a candle will not diminish no matter how many come to light their torches by it." That is the spirit which should pervade all medical men.

The question of marriage often takes prominent hold of the young doctor's mind and sometimes of the student's. In fact a wife is essential if one wishes to live in several succeeding generations. The value of the woman is gauged by the custom of the country in which she lives. In South Africa the price of a wife is two cows, or a box of cartridges and six needles; at Korak an old pair of shoes; in Tartary, Asia, her weight in butter; Meshins, Asia, from a hog to ten oxen; Ceylon, a box of matches; Navajo, New Mexico, upwards of twelve horses; Ungara, South America, merchandise on the installment plan; in Greenland, a knife; Germany and Austria require a dowery about equal to the grooms' property; England, all a woman can bring over from America. In the United States the tables are turned. The man is often considered valueless.

For this reason a few definite and absolute rules may govern should emergency arise. These are: Never marry above your station, financially, physicially or mentally. Your inferiority is called to your attention semi-occasionally, lest you forget.

Don't look too far beneath you for a spouse, she will surely drag you down to her level. Always seek your equal and assist your wife as you progress and be ever happy afterwards. Perhaps it is better not to marry while yet an undergraduate, unless you have a good quantity of loose dollars lying around and do not know how to hold on to them. Do not marry in haste or you may repent at leisure. In such a case provide for alimony in advance. Never take up with an ambitious woman; society will absorb the time you need for study. Above all I pray you do not remain a bachelor for you may be without issue and all the good there is in you will be everlastingly lost to mankind. Before procuring a license see to it that your bride is not jealous, talkative or quarrelsome. If you are trapped into such a bargain listen to her orphic sayings but ask God to be kind to you during the siege and final bombardment, but after her demise you might perhaps use this epitaph:

Here lies my wife  
A sad slattern and shrew;  
If I said I regret it  
I should lie too.

It is also well not to marry for spite; you only disfigure your own conscience.

In the class of '84 a tall, lank, thin, and otherwise unhappy looking young man failed to receive the requisite percentage which would, under the law, give him the privilege to become a member of this honorable profession. He did not commit suicide nor try another term. Far worse, to spite the faculty, he went straightway and got married and that was the last history ever recorded—he passed into oblivion.

Before leaving this important subject look about you. The profession is overcrowded with bachelors. Why? They have lost their opportunities. The first few years of practice money came in slowly, hardly enough to support one, let alone two. After a while things became more prosperous but age and mature judgment also advanced and the poor lone man saw nothing but visions of ambitious mamas or high-spirited widows and he sank back in despair, ergo the lone bachelor. However, may they not by single blessedness invite paresis at 40 and necessitate chloroform at 60?

Some good matches I have known eminated from the sick room. There are so many things a nurse observes that it finally becomes a habit with her to notice everything going on around her patient. After a few months the spell, which caused her to seek the hospital, leaves her and she comes to; her clinical work is used as a stepping stone to something better; she aspires to the title of Mrs. Doctor,—most maidens do. Now she selects the most plyable to her congenial spirit among the six or eight internes and lets him know, in a sort of surreptitious way, that she is it. During her three years as undergraduate she has had ample opportunity to study the kinks in the other internes whose diplomacy differs widely from those of Witte and Komura. After the nuptials she makes good use of the experience thus gained in looking after the welfare of her husband as well as her own. Poor sinner, he never thought his sweetheart had learned all the ways and by-ways of the young doctor, nevertheless, it was the best investment he had ever made. Such marriages, I believe, have never proven a failure, in fact, the nurse sees to it that they don't.

In this country, 5,000 young doctors graduate each year. What becomes of them? Do they all practice medicine? Oh, no. The output of the different colleges has been carefully watched for 30 years and it was found that but one--seventh practice the first ten years. After that, but 50 per cent. of those remain. Many find they are not fitted for medicine, others become teachers, some study for pleasure and some because they like the title. Therefore those who remain have ample room. Competition will never thwart their ambitions.

As undergraduates, never seek a large medical college; great opportunities are enjoyed in the smaller school with large clinical material. Attend post-graduate classes after some years of practice and continue to attend clinics in different cities of the world from year to year until you have become master of the art.

Attach yourself to national, state or local societies which meet frequently to discuss means to relieve the suffering and prevent disease. In this we differ widely from the lawyer. Who ever heard of the State Bar Association discussing the question, "How to prevent litigation?"

The medical department of the University of Buffalo since 1846 has had many great men in its faculty. Among them were James P. White, Austin Flint, Frank H. Hamilton, Julius F. Miner, the bold surgeon, whose praises were sung in England, Edward M. Moore, the brilliant orator, whose lectures were worth while going miles to hear, the lamented and revered Thomas F. Rochester and many others.

Much could be said of the present faculty, but grim custom forbids eulogising the living. However, their deeds speak for themselves. You may profitably imitate them and thereby become a credit to yourself and an honor to your Alma Mater.

85 NORTH PEARL STREET.

